# Effect of gut microbiota on α‐amanitin tolerance in *Drosophila tripunctata*

**DOI:** 10.1002/ece3.6630

**Published:** 2020-08-11

**Authors:** Logan H. Griffin, Laura K. Reed

**Affiliations:** ^1^ Department of Biological Sciences University of Alabama Tuscaloosa AL USA

**Keywords:** alpha‐amanitin, amatoxin, detoxification, *Drosophila*, gut microbiota, symbiosis

## Abstract

The bacterial gut microbiota of many animals is known to be important for many physiological functions including detoxification. The selective pressures imposed on insects by exposure to toxins may also be selective pressures on their symbiotic bacteria, who thus may contribute to the mechanism of toxin tolerance for the insect. Amatoxins are a class of cyclopeptide mushroom toxins that primarily act by binding to RNA polymerase II and inhibiting transcription. Several species of mycophagous *Drosophila* are tolerant to amatoxins found in mushrooms of the genus *Amanita*, despite these toxins being lethal to most other known eukaryotes. These species can tolerate amatoxins in natural concentrations to utilize toxic mushrooms as larval hosts, but the mechanism by which these species are tolerant remains unknown. Previous data have shown that a local population of *D. tripunctata* exhibits significant genetic variation in toxin tolerance. This study assesses the potential role of the microbiome in α‐amanitin tolerance in six wild‐derived strains of *Drosophila tripunctata*. Normal and antibiotic‐treated samples of six strains were reared on diets with and without α‐amanitin, and then scored for survival from the larval stage to adulthood and for development time to pupation. Our results show that a substantial reduction in bacterial load does not influence toxin tolerance in this system, while confirming genotype and toxin‐specific effects on survival are independent of the microbiome composition. Thus, we conclude that this adaptation to exploit toxic mushrooms as a host is likely intrinsic to the fly's genome and not a property of their microbiome.

## Graphical abstract

Several species of mycophagous *Drosophila* are tolerant to amatoxins found in mushrooms of the genus *Amanita*, despite these toxins being lethal to most other known eukaryotes. This study assesses the potential role of the microbiome in α‐amanitin tolerance in six wild‐derived strains of *Drosophila tripunctata*. Our results show that the microbiome does not contribute to toxin tolerance in this system, while confirming genotype and toxin‐specific effects on survival independent of the microbiome.

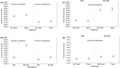

## 1 INTRODUCTION

The secondary metabolites of plants and fungi are often the medium through which they interact with animals (Mithöfer & Boland, 2012). Particularly, these compounds act as toxins that deter feeding or disrupt physiological processes and metabolism (Mithöfer & Boland, 2012). In evolutionary response to this phenomenon, herbivores have developed various mechanisms to defeat plant and fungal chemical defenses (Hammer & Bowers, 2015), which include metabolic degradation, target site insensitivity, metabolic sequestration, increased rate of secretion, and feeding behavior alteration to bypass ingesting high concentrations of toxins (Després, David, & Gallet, 2007). Additionally, many animals utilize their natural microbiota to detoxify harmful compounds they encounter in their environment (Ceja‐Navarro et al., 2015; Hammerbacher et al., 2013; Kohl, Weiss, Cox, Dale, & Dearing, 2014). Many species of *Drosophila* are generalists on Basidiomycota mushrooms, where they can spend their entire life cycle (Hackman & Meinander, 1979; Jaenike, 1978a, 1978b; Kimura & Toda, 1989; Lacy, 1984; Scott‐Chialvo & Werner, 2018; Shorrocks & Charlesworth, 1980). These insects are also among the only known eukaryotes to tolerate the amatoxins found in many of the mushrooms of this group (Jaenike, 1985; Jaenike, Grimaldi, Sluder, & Greenleaf, 1983; Scott‐Chialvo & Werner, 2018). Chief among these toxins is α‐amanitin, which acts by binding to primarily RNA polymerase II (RNAP II) and inhibiting transcription (Lindell, Weinberg, Morris, Roeder, & Rutter, 1970). Although α‐amanitin is extremely lethal even in small doses to nearly all eukaryotes, mycophagous *Drosophila* can tolerate this toxin in natural concentrations and utilize toxic mushrooms as larval hosts. While the mode of action of the toxin is well understood, the physiological mechanism of the flies' tolerance is not. However, we do know that this tolerance is not due to mutation in its target, RNAP II (Jaenike et al., 1983; Stump, Jablonski, Bouton, & Wilder, 2011). Additionally, the inhibition of glutathione S‐transferases results in no loss of tolerance, and inhibition of cytochrome P450s results in the loss of tolerance in only some species (Stump et al., 2011).

We hypothesize that larvae of these species metabolize, detoxify, and egest the amatoxins found in their diet, but these animals do not face the peril of plant and fungal chemical defenses alone but together with the microbes that inhabit their digestive tracts. Several animal species are known to exploit their gut microbial communities to enhance energy extraction and detoxification of compounds that they encounter in their natural diets (Douglas, 2009; Hammer & Bowers, 2015). Recently, the function of these microbes has been found to also include the manipulation and degradation of plant and fungal secondary metabolites (De Fine Licht & Biedermann, 2012; Hammerbacher et al., 2013; Kohl et al., 2014). However, these symbiotic gut microbes can also be vulnerable to some plant or fungal toxins (Kim, 2008) and could potentially be their primary target (Mithöfer & Boland, 2012). For example, the coffee borer beetle (*Hypothenemus hampei*) is only able to detoxify the high concentrations of caffeine found in coffee beans due to its symbiotic relationship with one bacterial species (*Pseudomonas fulva*) found in its gut (Ceja‐Navarro et al., 2015), and cabbage root fly larvae (*Delia radicum*) are able to detoxify isothiocyanates via degradative enzymes encoded in the gut microbiome (Welte et al., 2016). It stands to reason that the microbes that inhabit the intestinal tracts of mycophagous *Drosophila* species could contribute to the mechanism(s) that confer amatoxin tolerance. Prior studies have not ruled out the potential for the gut microbiota to confer or contribute to amatoxin tolerance in mycophagous *Drosophila* (Jaenike et al., 1983; Stump et al., 2011). In this study, we investigate the role of the bacterial gut microbiota in amatoxin tolerance.

## 2 METHODS AND MATERIALS

### 2.1 Insect specimens

*Drosophila tripunctata* specimens were obtained from a local population (University of Alabama campus, Tuscaloosa, AL, USA) by aspiration from mushroom and banana traps located within an area of approximately 100 square meters over the course of one month in November 2015. Single wild inseminated females were then mated with only full siblings to establish isofemale lines and were propagated for several generations. Lines used in the study were *Wolbachia*‐free as determined by a PCR test with *Wolbachia‐*specific primers. All flies and experimental treatments were maintained in routine culture at 22°C under a 12h:12h (L:D) photoperiod at 50% humidity. Stocks were maintained in standard fly vials on a diet of Carolina Biological Formula 4‐24 Instant Drosophila Medium supplemented with fresh white mushroom (*Agaricus bisporis*) purchased from a grocer.

### 2.2 Tolerance assays

Fifteen *D. tripunctata* first instar larvae were transferred into 5 ml sterilized glass vials containing 250 mg of 73.5% Carolina Biological Formula 4‐24 Instant Drosophila Medium and 26.5% ground freeze‐dried *A. bisporis* mushrooms hydrated with 1 ml water. Through pilot studies of varying larval density and food volume, we have found these control conditions to be optimal for raising healthy *D. tripunctata* larvae. The food for the antibiotic‐treated flies was rehydrated with 1 ml of a mixture of 50 µg/ml tetracycline, 200 µg/ml rifampicin, and 100 µg/ml streptomycin (Sigma) dissolved in sterile deionized water. This antibiotic regime was adopted to target a large variety of fly commensal bacteria (Ceja‐Navarro et al., 2015), since single antibiotic treatments are not adequate to target all commensal bacteria (Heys et al., 2018; Ridley, Wong, & Douglas, 2013). The flies receiving the toxin treatment also received a dose of 50 µg/ml of α‐amanitin in their food. Fifty µg/ml of α‐amanitin was determined to be a concentration that would guarantee death in amatoxin intolerant flies, while causing tolerant flies no harm under standard conditions (Jaenike, 1985; Jaenike et al., 1983; Stump et al., 2011). Five replicates of 15 first instar larvae were generated for each of the six isofemale lines for each of the four treatments (control, only antibiotics, only toxin, antibiotics, and toxin). The number of individuals surviving to pupation and adulthood was scored over a four‐week period, after which the experiment was terminated. Vials were checked daily to record larval development to pupation and eclosion. The number of individuals surviving to adulthood was recorded and analyzed, but survival to pupation was considered the most direct index of larval performance. All manipulations were performed under a laminar flow hood using aseptic technique. Adults that emerged from the assays were collected within 24 hours of eclosion by anesthetization by CO_2_. They were then frozen in PBS at −20°C for approximately three weeks where upon they were thawed, separated by sex, dried overnight in a 70°C oven, and weighed. Each fly was weighed individually to a precision of 0.01 mg.

### 2.3 Confirmation of microbiome knockdown

Dechorionation of eggs followed by transfer of the eggs to sterile food is often used to produce axenic fly cultures (Koyle et al., 2016; Ridley et al., 2013). However, we found that *D. tripunctata* was not sufficiently tolerant to dechorionation to feasibly conduct experiments. Furthermore, the laboratory diet used to rear *D. tripunctata* is not able to be autoclaved due to burning and caramelization of sugars. Single antibiotic treatments usually cannot entirely eliminate microorganisms from the gut of *Drosophila* (Ridley et al., 2013), but we did find that the antibiotic regimen outlined above was able to reduce bacterial load by >99%. Microbiome knockdown was confirmed by culturing surface sterilized mechanically homogenized larvae on standard methods agar containing 15.0 g agar, 5.0 g casein peptone, 2.5 g yeast extract, and 1.0 g glucose per liter for 72‐hr at 37°C using the pour plate method under aseptic conditions (Figure S1). After feeding for 72‐hr, three replicates of 15 larvae were aseptically collected and pooled, surface sterilized in 70% ethanol, then mechanically homogenized in 1 ml PBS. The entire undiluted homogenate was then pipetted into a sterile 90 mm × 15 mm petri dish where 25 ml of 45–50°C standard methods agar was then poured. The plates were then gently swirled to mix the larval homogenate and media and allowed to solidify in a laminar flow hood. Plates were incubated at 37°C degrees for 72 hr prior to inspection for bacterial growth. We did not specify a limit of detection because the plates represent undiluted samples. Since undiluted antibiotic‐treated samples gave an average of <1 CFU/ml, we did not dilute them further. As a result, we chose to also leave the no‐antibiotic samples undiluted, which produces an uncountable number of colonies. While not explicitly quantifiable, these results demonstrate a clear and substantial qualitative difference in bacterial load between antibiotic‐treated and no‐antibiotic samples.

Additionally, qPCR analysis was done to quantify differences in concentrations of 16S rRNA gene sequences (F: AGAGTTTGATCCTGGCTCAG, R: CTGCTGCCTCCCGTAGGAGT) between antibiotic and normal treatments. DNA was extracted using the DNeasy Blood and Tissue Kit (Qiagen) following manufacturer protocols. qPCR analysis showed that there was an approximately 180‐fold decrease in 16S rRNA gene sequences (Antibiotic Ct = 29.28; Nontreated Ct = 21.785; *p* = 5*10^–8^) in larvae treated with antibiotics for three days compared to untreated larvae (Table S10).

### 2.4 Data analysis

All statistical analyses were performed using JMP 14. Survival data were analyzed with logistic regression in the JMP fit model function, estimating the chi‐squared statistic using the likelihood ratio. Initial models assessed genetic line, toxin treatments, and microbiome treatments as main effects and estimated all the interaction effects among the main effects. To determine which lines were responsible for significant line‐by‐microbiome or line‐by‐toxin treatment interaction effects, logistic regression was run on each line and on the combination of line and microbiome treatment individually. Development time to pupation and adulthood was analyzed using nonparametric Kaplan–Meier method grouping by antibiotic treatment, toxin treatment, or both. Significance of the *p*‐values following Bonferroni correction for multiple testing is provided in the supplemental tables. Weight data were analyzed on each sex separately with standard least squares using genetic line, toxin treatment, and microbiome treatment as main effects. The microbiome‐by‐toxin interaction effect was also estimated. There was insufficient sample size for weight to estimate other interaction terms. Survival data are graphed as the proportion surviving ± 1 standard error (*SE*) where *SE* is estimated as $\sqrt{\left(\left(p\right)\times \left(1‐p\right)/n)\right)}$, where *p* in the proportion surviving. Summary statistics for the measured phenotypes can be found in Tables S1–S3.

## 3 RESULTS

The experiments described here quantified the survival rate to adulthood of antibiotic‐treated and conventional *Drosophila tripunctata* reared on diets containing 50 µg/ml of α‐amanitin. We hypothesized that the bacterial gut microbiota may be contributing to the toxin‐tolerant phenotype displayed by *D. tripunctata* on the basis that no physiological mechanism for amatoxin tolerance has yet been determined.

### 3.1 Tolerance assays

**FIGURE 1:**
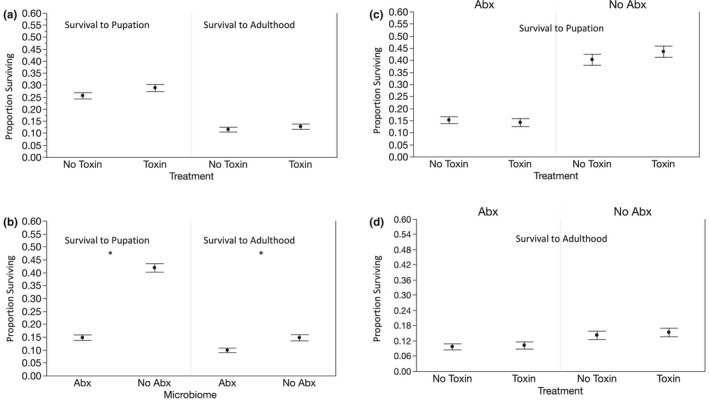
Mean proportion survival to pupation and adulthood across all genetic lines tested. Survival by toxin treatment (a) showed no significant effect, while survival across antibiotic treatments (b) show significant reductions of in survival for antibiotic‐treated flies. No significant interaction between toxin and antibiotic treatment was observed for either antibiotic (c) or toxin (d) treatment. Each error bar is constructed using one standard error from the mean

**FIGURE 2:**
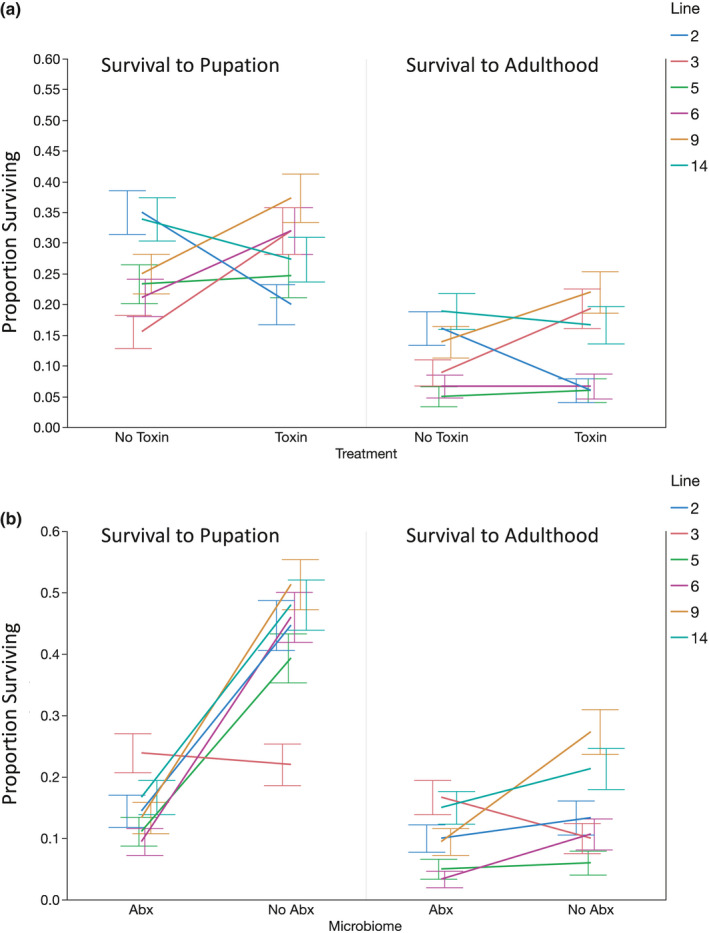
Mean proportion survival to pupation and adulthood from pupal stage across all genetic lines by toxin treatment (a) and antibiotic treatment (b). Each error bar is constructed using one standard error from the mean

**FIGURE 3:**
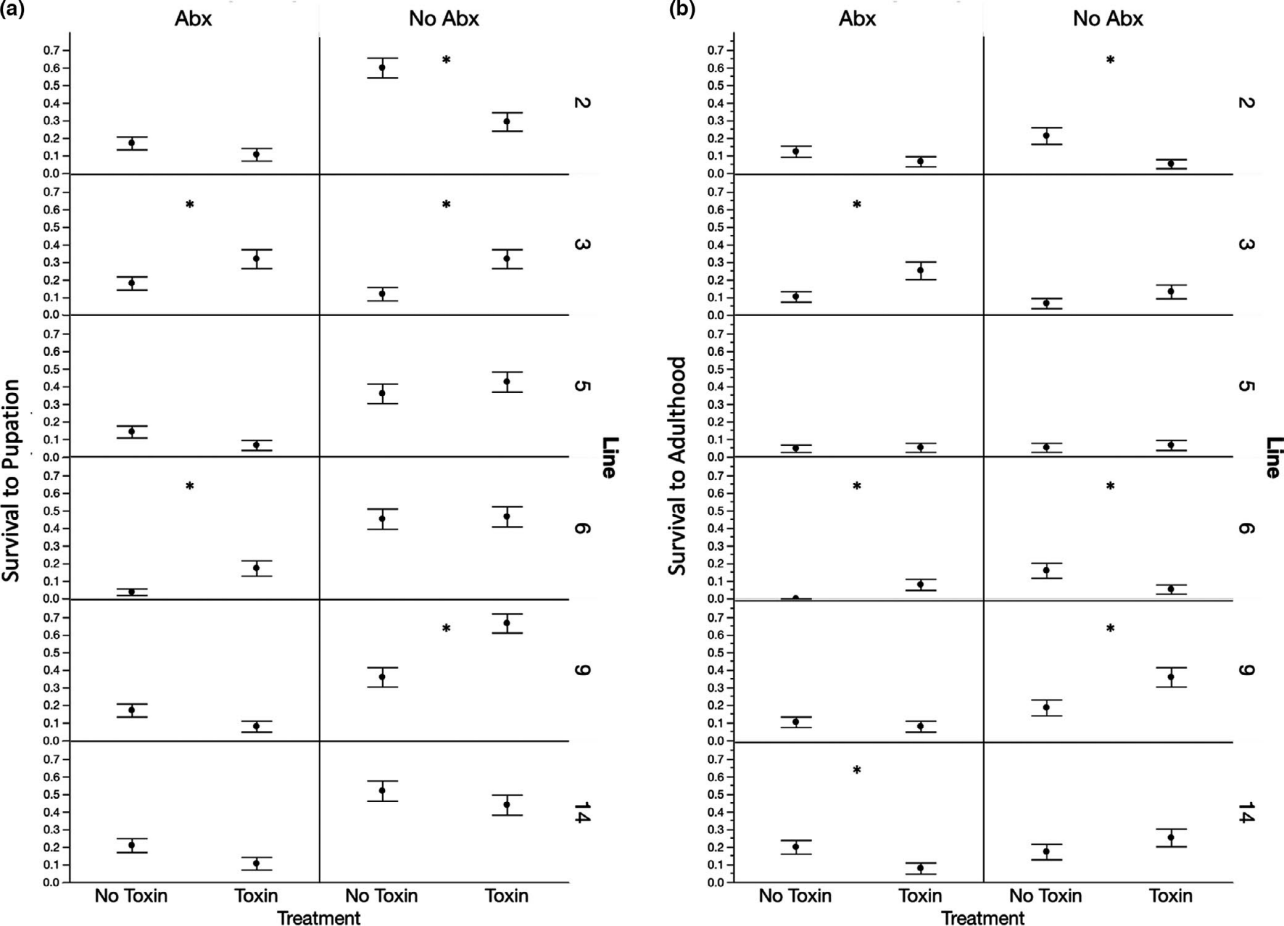
Mean proportion survival to pupation (a) and adulthood (b) versus antibiotic treatment, toxin treatment, and genetic line. Each error bar is constructed using one standard error from the mean. *Significance *p* < .05 in the logistic regression of toxin presence/absence within genotype and antibiotic treatment as see in Table S6

Survival to pupation and survival to adulthood were not significantly impacted by the main effects of genetic line or toxin presence (Table 1 and Figure 1a). While the reduction of the bacterial load did have a significant impact on survival independent of toxin presence (Table 1 and Figure 1b), with higher survival when the microbiome was normal, which has also been observed in other microbiome studies of *Drosophila* (Wong, Dobson, & Douglas, 2014). Of particular interest to the central question of this study was whether there was an interaction between the antibiotic treatment and the presence of toxin on the survival traits, and there was no significant interaction effect between these two effects (Table 1 and Figure 1c,d). This implies that the bacterial microbiome is probably not critical to toxin tolerance in this species. We did observe significant interaction effects between toxin tolerance and genetic line on survival (Table 1 and Figure 2a), as well as significant interactions between the antibiotic treatment and genetic line on survival (Table 1 and Figure 2b). Note that the significant interaction between genetic line and antibiotic treatment for survival to pupation does disappear if genetic line “3” is removed from the analysis, but this does not change the relative significance of the other effects in the models (Table S4). The interaction effects between genetic line and toxin presence and the antibiotic treatment show that there is genetic variation within this species for the impact of these environmental factors on the survival. The three‐way interaction between genetic line, the presence of toxin, and antibiotic treatment is also significant for both survival traits (Table 1). Of note, the significant interaction effect on survival between genetic line and toxin presence was observed both with and without antibiotic treatment, suggesting that the differences in toxin tolerance among genetic lines are probably not mediated by the robust presence of the bacterial microbiome alone (Figure 3). When inspected closely, in the presence of antibiotics (reduction of microbiome), survival to pupation was higher in the presence of toxin (Figure 3, Tables S5 and S6) for two genetic lines (“3” and “6”). Conversely of the three lines that showed an impact of the toxin on survival to pupation in the presence of the microbiome, two showed increased survival on toxin and one showed decreased survival (Figure 3, Tables S5 and S6). Only one genetic line showed any evidence consistent with the hypothesis that the microbiome might help to mediate toxin tolerance, line “14,” which showed reduced survival to adulthood when exposed to both the toxin and antibiotics (Figure 3).

**TABLE 1:** Logistic regression model effects and significant values for survival phenotypes to pupation and adulthood

Phenotype	Effect	DF	Likelihood ratio (Chi‐square)	*p*‐value
Survival to Pupation	Line	5	7.100863891	.2132
Survival to Pupation	Toxin	1	0.163293122	.6861
Survival to Pupation	Microbiome	1	167.1890045	<.0001
Survival to Pupation	Line*Toxin	5	37.12552217	<.0001
Survival to Pupation	Line*Microbiome	5	50.42904534	<.0001
Survival to Pupation	Toxin*Microbiome	1	1.61785934	.2034
Survival to Pupation	Line*Toxin*Microbiome	5	24.10883831	.0002
Survival to Adulthood	Line	5	42.28944085	<.0001
Survival to Adulthood	Toxin	1	1.768604174	.1836
Survival to Adulthood	Microbiome	1	11.10332103	.0009
Survival to Adulthood	Line*Toxin	5	21.21703667	.0007
Survival to Adulthood	Line*Microbiome	5	23.34079246	.0003
Survival to Adulthood	Toxin*Microbiome	1	2.676768793	.1018
Survival to Adulthood	Line*Toxin*Microbiome	5	26.31790088	<.0001

### 3.2 Development time

**FIGURE 4:**
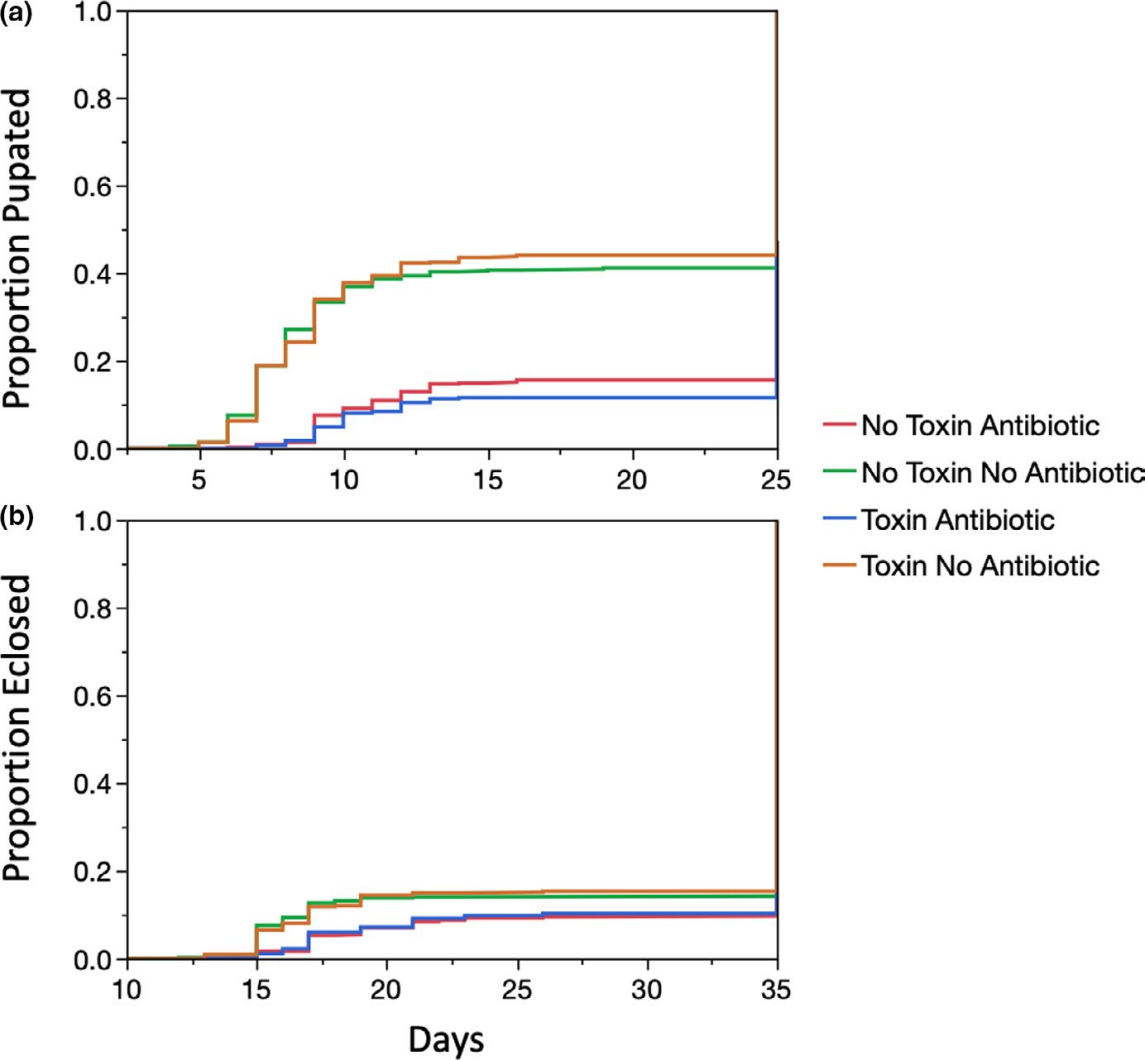
Development time increased by antibiotics but not by toxin. The proportion to pupate by time for those larvae that did reach pupation was significantly longer in the with antibiotic treatment (*p* < .0001) but was not influenced by the presence of toxin (a) The proportion to eclosion as adults by time for those larvae that did reach adulthood was significantly longer with the antibiotic treatment (*p* < .0001) but the presence of toxin had no influence on the time needed to develop to adulthood (b)

In addition to tracking survival, we measured the days until pupation and eclosion for the larvae. Of those larvae that did succeed in pupating or eclosing, we found that they took significantly longer to reach pupation and eclosion in the presence of the antibiotic (*p* < .0001, Log‐Rank test, Figure 4), but that there was no significant difference in development time between toxin and no toxin flies within the antibiotic treatment (Table S7). While within some genetic lines (“2”,“9”,“14”) toxin treat flies developed to pupae more quickly than nontoxin‐treated flies (Tables S7 and S8), and one genetic line (“6”) showed slower development to both pupation and eclosion on toxin in the absence of antibiotics, no genetic lines showed any evidence consistent with the hypothesis that the microbiome might provide toxin tolerance for development time (Table S7).

### 3.3 Adult weights

Males and females were analyzed separately as males are consistently lighter than females in flies. We did not find any significant differences in adult dry weight as an effect of genetic line, antibiotic, and toxin treatment (Figure S2 and Table S9). The lack of significant effects could be driven by survivor bias, only those that survived to adulthood were weighed, and/or by a relatively small sample size for weights also due to the low rate to survival to adulthood. However, there was no evidence in the weight data that suggests that an increased sample size would change the conclusion of a lack of a microbiome‐by‐toxin interaction effect (Table S3 and Table S9).

## 4 DISCUSSION

Plants and mushrooms have evolved to synthesize an elaborate array of protective chemicals to ward off phytophagous and mycophagous animals (Fraenkel, 1959; Howe & Jander, 2008; Martin, 1979). The ability to avoid the toxic effects of these defensive compounds found in their food is critical for survival of many animals and is a key adaptation in their evolution (Ehrlich & Raven, 1964). Avoiding the toxicity of these compounds can be achieved in various ways such as behavioral avoidance of tissues containing toxins, target site insensitivity, or metabolic degradation, sequestration, and/or excretion (Després et al., 2007; Wilson & Hooser, 2001). Several species of mushrooms of the genus *Amanita* contain significant amounts of cyclopeptide toxins in their tissues (Li & Oberlies, 2005). There are 17 known species of mycophagous *Drosophila* that use mushrooms that contain cyclopeptides toxins as developmental hosts (Scott‐Chialvo & Werner, 2018). No clear physiological mechanism for this tolerance has yet been identified, although it is clear that it is not due to target site insensitivity or behavioral avoidance (Jaenike et al., 1983). It is well understood that the interactions between metazoans and their microbiota are crucial for many of the host's phenotypes and is substantially affected by microbial composition, host genotype, and the environment (Faith, Mcnulty, Rey, & Gordon, 2011; Gevers et al., 2012; Kau, Ahern, Griffin, Goodman, & Gordon, 2011; Turnbaugh et al., 2009). Microbial symbionts, especially those of the gut, are known to play crucial roles in several physiological processes such as in development, growth, fecundity, immune response, pesticide resistance and detoxification, and amino acid metabolism (Berasategui et al., 2017; Ceja‐Navarro et al., 2015; Coon, Vogel, Brown, & Strand, 2014; Lee et al., 2017; Welte et al., 2016; Yamada, Deshpande, Bruce, Mak, & Ja, 2015). This study investigated the role of the bacterial gut microbiota in amatoxin tolerance in the mycophagous *Drosophila* species *D. tripunctata*. We report the first experimental evidence suggesting that the gut microbiome is likely not centrally responsible for conferring α‐amanitin tolerance to this species. If the microbiome was indeed conferring toxin tolerance to the flies, we would expect to see a significant reduction in survival in the presence of toxin in the groups treated with the antibiotic regimen when compared to groups that were treated with the toxin alone.

These data indicate that in *D. tripunctata*, the bacteria in the gut likely play little to no role in conferring tolerance to α‐amanitin. Given α‐amanitin's mechanism of action of binding to RNAPII, which is solely eukaryotic, it is reasonable to think that bacteria would not be negatively affected by this toxin. Amatoxins are rich in carbon and nitrogen, and it is therefore reasonable to hypothesize that some bacteria may metabolize amatoxins for their own benefit; however, to date no study has investigated the bacterial capability for degrading or metabolizing α‐amanitin or other amatoxins, likely due to the high financial cost of the toxin. Our results are consistent with the idea that one or more physiological mechanisms innate to the flies likely exist that confers toxin tolerance, and further investigation is required to elucidate that mechanism. We cannot, however, rule out the possibility that other nonbacterial commensal gut microbes (e.g., yeasts) may be contributing to the tolerance mechanism. Although we have demonstrated that significantly reducing bacterial load does not change toxin tolerance in *D. tripunctata*, further work is needed to determine whether the microbiome may play a role in other toxin tolerant mycophagous *Drosophila* species. Martinson, Douglas, and Jaenike (2017) surveyed four sympatric, amatoxin tolerant species (*D. falleni*, *D. recens*, *D. neotestacea*, and *D. putrida*) of wild mycophagous *Drosophila* that shared a common diet of decaying mushrooms. Despite the deep phylogenetic divergence of these species, they shared very similar microbiomes which consistently contained species that were either in low abundance or undetectable in their environment. Thus, we believe it is possible that those species also share a common microbiome with *D. tripunctata*. Due to the generation of isofemale lines and their maintenance in the laboratory, it is worth noting that a bottlenecking effect on the microbiota community may have been present prior to experimentation, which may have eliminated some of the native bacteria. However, if this bottlenecking did occur and eliminated the bacteria responsible for conferring tolerance, it would have been detected as a decrease in toxin tolerance in flies even in the absence of antibiotics.

Our results do not support a bacterial mechanism being primarily responsible for amatoxin tolerance in *Drosophila tripunctata,* and future studies should work to identify what the mechanism of tolerance might be, including consideration of the nonbacterial components of the gut microbiome. Further work should also be done to thoroughly characterize the potential role of the microbiome in other mycophagous species as well.

## CONFLICTS OF INTEREST

The authors declare no conflicts of interest.

## AUTHOR CONTRIBUTION

**Logan H. Griffin:** Conceptualization (equal); Data curation (equal); Formal analysis (supporting); Investigation (lead); Methodology (lead); Project administration (lead); Visualization (supporting); Writing‐original draft (lead); Writing‐review & editing (equal). **Laura K. Reed:** Conceptualization (equal); Data curation (equal); Formal analysis (lead); Funding acquisition (lead); Methodology (equal); Project administration (equal); Supervision (lead); Visualization (lead); Writing‐original draft (equal); Writing‐review & editing (equal).

## Supporting information

Fig S1‐S2

Table S1‐S10

## Data Availability

Supplemental data are available on the Dryad data repository at https://doi.org/10.5061/dryad.vdncjsxrp.
