# Supplementary material for: Effect of gut microbiota on α‐amanitin tolerance in Drosophila tripunctata
Source: Ecol Evol. 2020 Aug 11;10(17):9419–27. doi: 10.1002/ece3.6630 (PMC7487225; doi:10.1002/ece3.6630)
Supplement: Supplementary file 1 — Fig S1‐S2 [file ECE3-10-9419-s001.docx]

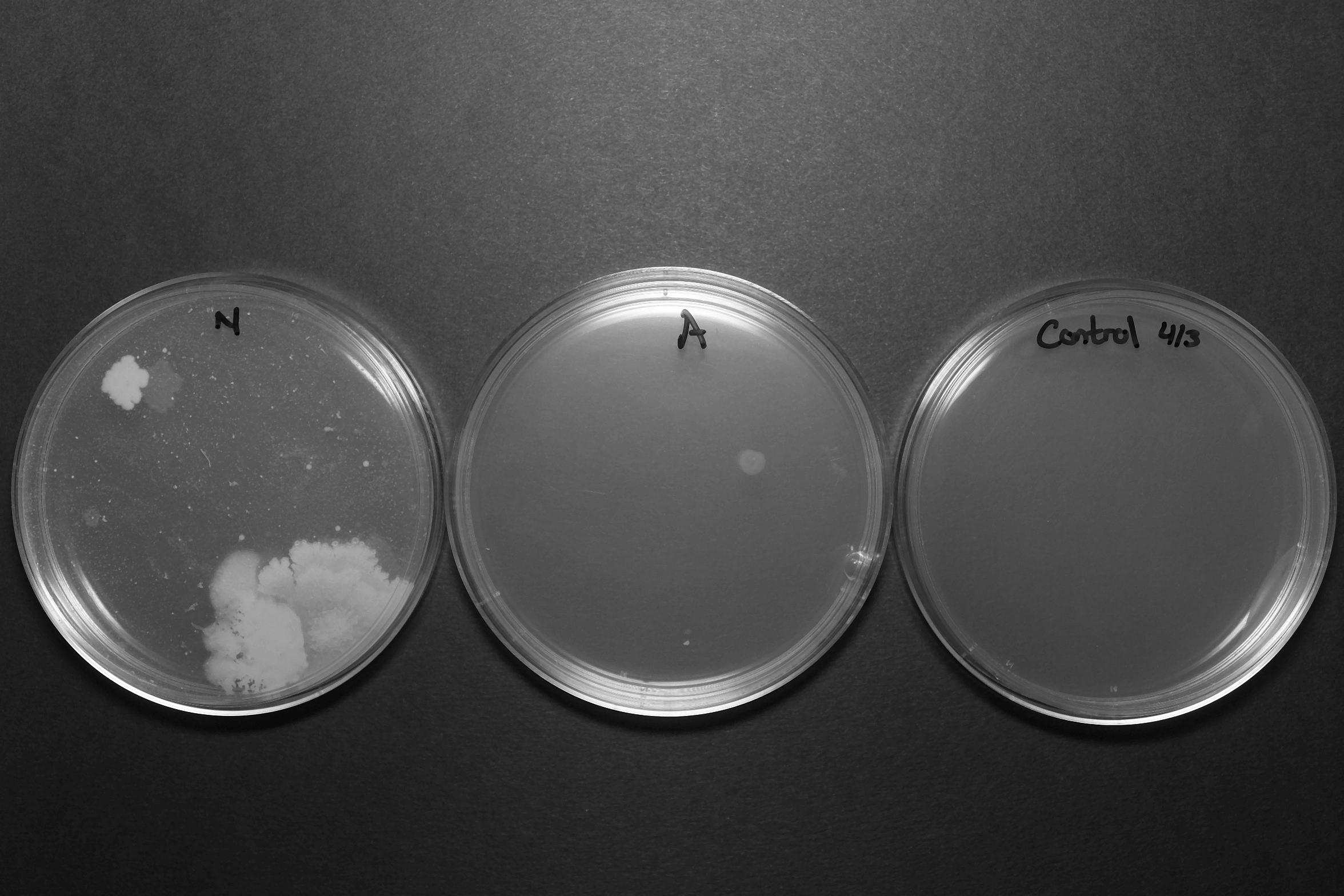


**Figure S1.** Microbiome knockdown was confirmed by culturing surface sterilized mechanically homogenized larvae on standard methods agar for 72-hours at 37**°**C using the pour plate method under aseptic conditions. Each plate was done in triplicate. (**Left**) Culture of non-antibiotic treated homogenized larvae. (**Center**) Culture of homogenized larvae from antibiotic-treated larvae. (**Right**) Negative control containing only agar.

**Figure S2.** Mean dry weight vs. antibiotic & toxin treatment by genetic line. Results are averaged across both sexes. Each error bar is constructed using one standard error from the mean.
